# Advances in Neuroimmunology: From Bench to Bedside

**DOI:** 10.1155/2014/812847

**Published:** 2014-01-19

**Authors:** Cristoforo Comi, Umberto Dianzani, Filippo Martinelli Boneschi, Daniel L. Menkes

**Affiliations:** ^1^Department of Translational Medicine, Section of Neurology, University of Eastern Piedmont Amedeo Avogadro, 28100 Novara, Italy; ^2^Interdisciplinary Research Center of Autoimmune Diseases (IRCAD), University of Eastern Piedmont Amedeo Avogadro, 28100 Novara, Italy; ^3^Department of Neurorehabilitation and INSPE, San Raffaele Scientific Institute, 20132 Milan, Italy; ^4^Department of Neurology, Oakland University William Beaumont School of Medicine, Royal Oak, MI 48073, USA

The understanding of the interactions between the immune and the nervous systems and the resultant therapeutic implications has expanded significantly in the last decade [1]. There have been significant developments in the field of neuroimmunology as new antibody-mediated disorders have been described and involvement of the immune system in the pathogenesis of neurodegenerative diseases has been established [2, 3]. These discoveries have led to novel and effective treatments, which have broadened our therapeutic options regarding neuroimmune disorders [4, 5].

The goal of this special issue was to address the translational aspects of neuroimmunology, “from bench to bedside,” in order to update clinicians on basic research discoveries that will have therapeutic clinical efficacy. Moreover, there was an emphasis on conditions that have undergone a systematic nosographic characterization which have resulted in therapeutic approaches with greater specificity. In this context, the paper entitled “*Immunotherapy of neuromyelitis optica*” provides a framework for understanding an antibody-mediated central nervous system demyelinating disease that has a different pathophysiology than multiple sclerosis (MS). This distinction is important as NMO responds to different immunomodulating agents than does MS.

The spectrum of pediatric MS has also been the focus of extensive nosographic revision in recent years, and diagnostic criteria have been recently revised by the International Pediatric Multiple Sclerosis Study Group (IPMSSG) [6]. The paper “*Pediatric multiple sclerosis: current concepts and consensus definitions” *offers a careful update on risk factors, clinical manifestations, diagnostic procedures, prognostic implications, and treatment of this increasingly frequent form of MS.

MS is a salient neuroimmunological disease for which the “bench to bedside approach” has provided the greatest therapeutic advances. Although there are more treatments for MS, the study of novel and less explored molecular pathways ought to provide relevant alternative targets. This concept is well expressed in the paper entitled “*Current understanding on the role of standard- and immuno-proteasomes in inflammatory/immunological pathways of Multiple Sclerosis*,” in which the authors describe the current knowledge on the potential role of proteasomes in MS and discuss the *pro et contra *of possible therapies for MS targeting proteasome isoforms.

Immune mediated diseases of the peripheral nervous system (PNS) are less studied than their “central” counterparts [7]. Nonetheless, important advances in both pathogenesis and treatment of inflammatory demyelinating neuropathies have been extensively evaluated in the articles authored by J. B. Weiner and P. Ripellino et al. The first publication entitled “*An update in Guillain-Barré Syndrome*” provides a comprehensive discussion of the current state of knowledge on acute inflammatory neuropathies from diagnosis to treatment. The second article, entitled “*Treatment of chronic inflammatory demyelinating polyneuropathy: from molecular bases to practical considerations,” *bridges the biological rationale of immunotherapy to clinical practice also in the context of pharmacoeconomics.

Finally, the paper entitled “*An update in the use of antibodies to treat glioblastoma multiforme*” reviews the current knowledge on an expanding field immunotherapy, which is expected to have a significant impact on the progression of these high grade gliomas.

The editors believe that you will agree that this special issue will prove to be highly valuable to basic scientists and clinicians alike.

*Cristoforo Comi* *Umberto Dianzani* *Filippo Martinelli Boneschi* *Daniel Menkes*
